# Influence of Rotational Speed on Isothermal Piston Compression System

**DOI:** 10.3390/e25040644

**Published:** 2023-04-12

**Authors:** Teng Ren, De-Xi Wang, Wei-Qing Xu, Mao-Lin Cai

**Affiliations:** 1 School of Mechanical Engineering, Shenyang University of Technology, Shenyang 110870, China; ren_teng@buaa.edu.cn; 2 School of Automation Science and Electrical Engineering, Beihang University, Beijing 100191, China; weiqing.xu@buaa.edu.cn (W.-Q.X.); caimaolin@buaa.edu.cn (M.-L.C.)

**Keywords:** isothermal piston, porous media, flow resistance, energy conservation

## Abstract

An isothermal piston is a device that can achieve near-isothermal compression by enhancing the heat transfer area with a porous media. However, flow resistance between the porous media and the liquid is introduced, which cannot be neglected at a high operational speed. Thus, the influence of rotational speed on the isothermal piston compression system is analyzed in this study. A flow resistance mathematical model is established based on the face-centered cubic structure hypothesis. The energy conservation rate and efficiency of the isothermal piston are defined. The effect of rotational speed on resistance is discussed, and a comprehensive energy conservation performance assessment of the isothermal piston is analyzed. The results show that the increasing rate of the resistance work increases significantly proportional to the rotational speed, and the proportion of resistance work in the total work increases gradually and sharply. The total work including compression and resistance cannot be larger than the compression work under adiabatic conditions. The maximum rotational speed is 650 rpm.

## 1. Introduction

The acceleration of industrialization and rapid growth of the global population have resulted in an exponential growth in energy consumption, particularly in the electricity sector [1,2]. Around 90% of the world’s energy demand is supplied by fossil fuels such as coal, oil, and natural gas [3,4]. This leads to environmental problems due to undesired gas emissions associated with fossil fuels. Greenhouse gases, such as carbon dioxide, methane, and nitrous oxide, are emitted in large quantities during the combustion process of fossil fuel, causing weather changes, sea level rise, the destruction of ecosystems, severe health problems, etc. [5]. Such climate changes and health threats to human beings have raised public attention in the world [6,7]. For this reason, all nations have adjusted their energy policies to minimize these problems. China has pledged that its carbon footprint will peak by 2030 and become carbon neutral by 2060. Although fossil fuels are not sustainable and result in severe environmental and health problems, they are still the basis for human survival and development for a long time in the future [8,9,10]. Renewable energy resources (RES) are the most promising alternatives to fossil fuels, but it is worth mentioning that the current realization of renewable energy resources has to overcome many challenges and barriers [11,12].

RES such as solar photovoltaic and wind energy have obstacles in terms of their connectivity to the power grid, mainly due to their intermittent nature [13]. In this regard, energy storage systems (ESSs) have been widely considered as an ultimate solution to smooth the renewable energy systems’ power generation scheme [14,15]. Therefore, the development of appropriate energy storage technologies is of great significance for the application of renewable energy resources [16,17].

Among other ESSs, including fuel cells, batteries, mechanical energy storage such as flywheels, super magnetic energy storage, etc., only compressed air energy storage (CAES) and pumped hydro energy storage (PHES) can be utilized for large-scale applications due to their advantage of having long discharge times [18,19]. There are vital obstacles that make PHES complicated, which include geological constraints in terms of providing adequate and massive rooms for reservoir constructions, as well as environmental concerns [18,20]. Conversely, CAES can store energy in the form of compressed air either in high-pressure vessels or underground or underwater reservoirs [21]. For large-scale CAES, compressed air is stored in the reservoirs, typically in forms of underground geology such as abandoned mines, depleted gas fields, rock caverns, and aquifers with sufficient porosity and permeability [18,22]. Therefore, CAES has fewer geological restrictions compared to PHES, and it will reduce construction and maintenance costs [19,23].

CAES is suitable for large-scale energy storage due to the high energy capacity and power rating. Nevertheless, its energy and power density are relatively low, for the lower utilization of thermal energy generated in the compression phase [24]. Therefore, due to the poor thermal efficiency, the energy efficiency of CAES is relatively low, with a range of 40% to 70% [21]. Isothermal compression can make for the minimum amount of compression work, for the compression heat of compressed air is transferred immediately during the compression phase. From this point of view, the isothermal compressed air energy storage (I-CAES) is another appropriate technology for large-scale energy storage. In I-CAES, the air temperature is kept nearly constant during the compression and expansion phases, and there is no need for fuel combustion [25]. The ideal round trip efficiency of I-CAES is 100% [26]. Though I-CAES is a promising emission-free technology with high efficiency that can be used to facilitate the integration of fluctuating renewable energy into the power grid, technical devices to realize isothermal compression/expansion are in research stages and no project of I-CAES has operated until now [27].

An isothermal piston is a kind of device that can achieve isothermal compression, and the performance of an isothermal piston was discussed in our former study [28,29]. It was verified that the isothermal piston can lower the air temperature significantly, but the resistance between the isothermal piston and liquid was introduced causing additional energy input. The frictional forces can be neglected at a relatively low speed (100 rpm). However, when the speed increases continually, the effect of flow resistance cannot be ignored. This paper analyses the influence of rotational speed on the isothermal piston, and explores the operation speed range of the isothermal piston compressor.

## 2. Isothermal Compressed Air Storage and Isothermal Piston

### 2.1. Isothermal Compressed Air Storage

Compressed air energy storage (CAES) is a kind of electric energy storage system, which can realize a large capacity and longtime storage [30]. Through the air compressor unit, CAES stores the excess electric energy in the off-peak period into the gas storage tank in the form of pressure potential energy of compressed air. When the demand for electricity increases, it releases the compressed air stored in the gas storage tank and converts the energy stored in the compressed air into electric energy through the turbine after heating, so as to meet the demand for electricity in the peak period. The CAES system can realize power production and consumption at different times, and realize the “peaking and valley filling” of the power grid to balance the power load, so as to improve the stability and reliability of the power grid.

**Figure 1:**
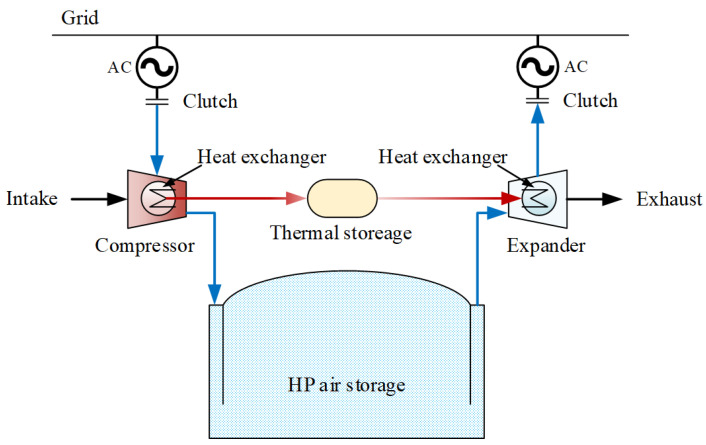
Isothermal compressed air energy storage.

Traditional CAES is operated under an adiabatic condition, where the air temperature increases significantly during air compression and the heat exchange is conducted outside the compressor. The compression heat is stored to improve energy efficiency. However, the total energy efficiency is limited due to the poor thermal efficiency. For isothermal compressed air energy storage, as shown in Figure 1, the heat of compression is transferred from the compression chamber to the thermal storage in the process of compression, so that the air temperature in the compression chamber remains unchanged as far as possible, and the compression work is minimized. When the stored energy needs to be utilized, the compressed air is released from the high-pressure (HP) air storage, and the compression heat of the compressed air stored during the compression phase is used to heat the released air in the expander. Therefore, isothermal compression is a promising method to improve the efficiency of CAES.

### 2.2. Isothermal Piston

**Figure 2:**
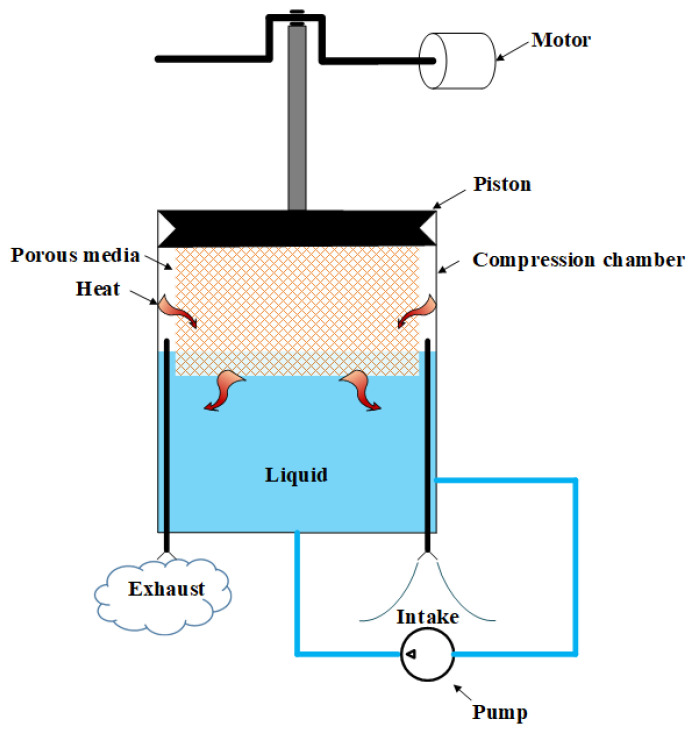
Isothermal piston.

The isothermal piston is a unique piston structure that makes sure the air temperature remains unchanged during compression. It is composed of a piston and porous media. As shown in Figure 2, the porous media is located beneath the piston and can move with the piston at the same time. A certain amount of liquid is placed at the bottom of the compression chamber to form a gas–solid–liquid coupling structure enhancing heat transfer. Due to its special sparse porous structure, porous media has a very large specific surface area, which greatly increases the heat transfer area between the gas, liquid, and solid, enhancing the heat transfer in the compression cavity greatly.

In the process of compression, the heat generated by the air compression is transferred to the porous media. With the process of compression, the porous media is gradually immersed in the liquid at the bottom of the compression chamber so that the heat absorbed by the porous media is eventually transferred to the liquid. Due to the large specific heat capacity of the liquid, the liquid temperature remains almost constant, and the heat transfer from the porous media to the liquid can be sustained. In practice, the liquid can be circulated through an external circulating pump to dissipate heat to the outside and keep the liquid temperature constant. All of this allows the heat conduction from the compressed air to the porous media to proceed continuously.

## 3. Resistance Model

It is assumed that the cross-sectional area of the porous media is equal to that of the compression cavity; that is, the porous media is completely filled with the compression cavity. The porous media has the characteristic of sparse porosity, and the internal micro pores are connected with each other irregularly, which makes the construction of the resistance model very complicated. In this study, a simplified modeling analysis of porous media was carried out based on a face-centered cubic structure.

The flow resistance between the porous media and liquid is caused by their relative motion. It is influenced by the structural characteristics of the porous media such as porosity, fluid properties, relative motion velocity, etc. The flow pressure gradient $\frac{dp_{liq}}{dL}$ is given by the Ergun surface cube model [31,32]:

(1) $$\frac{dp_{liq}}{dL}=\frac{\mu }{K}\cdot u+\rho \cdot C_{c}\cdot u^{2}$$

where d*p_liq_* is the flow pressure of the liquid during the porous media, the direction of d*L* is opposite to the piston motion, *μ* is the dynamic viscosity of the fluid, *ρ* is the fluid density, *u* is the relative velocity between the porous media and the fluid, *K* is the permeability of the porous media, and *C_c_* is the inertia coefficient of the porous media (or shape factor). *K* and *C_c_* are defined as

(2) $$K=\frac{\varepsilon^{3}d_{p}{}^{2}}{a{\left(1-\varepsilon \right)}^{2}}$$

(3) $$C_{c}=\frac{b\left(1-\varepsilon \right)}{\varepsilon^{3}d_{p}}$$

where *a* and *b* are constants related to the viscosity and inertia, respectively, *ε* is the porosity of the porous media, and *d_p_* is the equivalent spherical particle diameter, which can be defined as:

(4) $$d_{p}=\frac{6}{S_{v}}$$

where *S_v_* is the specific surface area of the porous media. Substituting Equations (2) and (3) into Equation (1) gives the classical Ergun equation

(5) $$\frac{dp_{liq}}{dL}=\frac{a{\left(1-\varepsilon \right)}^{2}\mu }{\varepsilon^{3}d_{p}{}^{2}}\cdot u+\frac{b\left(1-\varepsilon \right)}{\varepsilon^{3}d_{p}}\cdot \rho \cdot u^{2}$$

The resistance between the porous media and the air can be ignored when comparing the viscosity and density of air (17.9 × 10^−6^ Pa∙s and 1.18 kg/m^3^, respectively) and water (1.01 × 10^−3^ Pa∙s and 1 × 10^3^ kg/m^3^, respectively). In this work, the fluid resistance corresponds only to the liquid resistance.

## 4. Energy Consumption and Efficiency Calculation

The isothermal piston compression system was discussed in our former studies [28,29]. Compression work is the work produced by volume change. For an air compression system, the compression work is a key index to measure the energy consumption. The compression work can be obtained by integrating the air pressure over the volume:

(6) $$W=-\int_{V_{0}}^{V}p\cdot dV$$

where *p* is the air pressure, and *V*_0_ and *V* are the air volume at the beginning and end of compression, respectively. During the compression process, the volume of air decreases and the pressure increases. The negative sign indicates that the volume change of the gas is opposite to the pressure change trend, so the sign of the compression work is positive.

In particular, isothermal and adiabatic are two special types of gas compression processes. In the process of isothermal compression, the temperature of the air remains constant and the compression work input from outside is minimal. For a closed system, the isothermal compression work *W_iso_* can be expressed by Equation (7).

(7) $$W_{iso}=m_{air}\cdot R\cdot T_{0}\cdot \ln\frac{p}{p_{0}}$$

where *m_air_* is the mass of air in the closed system, *T*_0_ is the air temperature, *p*_0_ and *p* are the air pressure at the beginning and end of the compression process, respectively. Adiabatic compression refers to the compression process in which there is no heat exchange between the system and the outside, and all the compression work is converted into the internal energy of the air. For an adiabatic compression process, the pressure and temperature of the air are the highest at the same compression position; therefore, the compression work of adiabatic compression is the greatest in multiple forms of compression. The adiabatic compression work *W_adi_* can be expressed by Equation (8).

(8) $$W_{adi}=\frac{1}{\kappa -1}\cdot m_{air}\cdot R\cdot T_{0}\cdot \left[{\left(\frac{V_{0}}{V}\right)}^{\kappa -1}-1\right]$$

where *T*_0_ is the initial temperature, and *κ* is the adiabatic exponent, and usually makes it equal to 1. 4.

The viscosity and density of air are much less than those of water. Therefore, the flow resistance between the porous media and the air can be ignored during the operation of the isothermal piston. In this study, only the flow resistance between the porous media and the liquid is considered. When the piston runs at a constant speed, the flow pressure gradient remains constant, and the flow resistance changes linearly. Thus, the flow resistance work *W_por_* between the porous media and the liquid can be written as Equation (9).

(9) $$W_{por}=\frac{1}{2}\cdot \frac{dp_{liq}}{dL}\cdot l\cdot V_{cy}\cdot \left(1-\varepsilon \right)$$

where *l* is the length of the porous media, and *V_cy_* is the volume of the compression chamber.

For the isothermal piston air compression system, the total energy consumption *W_+por_* is comprised of the compression work and the flow resistance work:

(10) $$W_{+por}=W+W_{por}$$

From the perspective of energy consumption, compared with the adiabatic process, the more compression work saved, the better the energy conservation effect. Considering the additional flow resistance work, the energy conservation rate of the isothermal piston is defined as the following:

(11) $$\zeta =\frac{W_{adi}-W_{+por}}{W_{adi}}$$

In order to judge the degree to which the compression process approaches an isothermal state, the isothermal compression work is compared with the actual compression work and resistance work. The isothermal piston’s efficiency can be defined as the ratio of the isothermal compression work to the actual total work of the isothermal piston:

(12) $$\eta =\frac{W_{iso}}{W_{+por}}$$

The isothermal piston efficiency directly reflects the degree to which the system approaches an isothermal state. A higher isothermal piston efficiency represents a better heat transfer effect, and the efficiency approaches 100% when the air is compressed and approaches an isothermal condition. The efficiency is the lowest when compressed adiabatically, and the lower limit of isothermal piston efficiency is the ratio of isothermal compression work to adiabatic compression work, which is

(13) $$\eta_{\min}=\frac{W_{iso}}{W_{adi}}$$

## 5. Results and Discussion

### 5.1. Effect of Rotational Speed on Resistance

**Figure 3:**
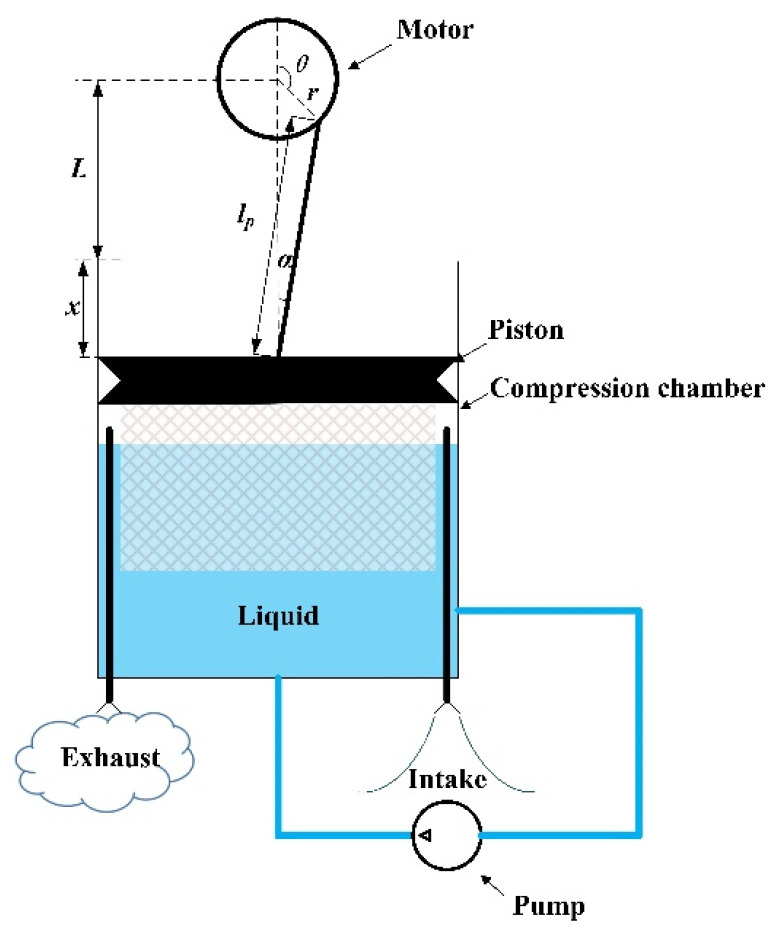
Isothermal piston operation diagram.

According to Equation (5), the compression speed has a great influence on the flow pressure gradient between the porous media and the fluid. In the application process of the compressor, the operation speed of the compressor is usually described by the motor speed. Therefore, it is necessary to convert the compression speed of the piston into the corresponding motor speed. As shown in Figure 3, the relation between the piston travel and the motor angle can be expressed as

(14) $$x=\sqrt{l_{p}^{2}-r^{2}\sin^{2}\theta }-r\cos\theta -L$$

where *x* is the piston travel, *θ* is the motor angle, *l_p_* is the length of the connecting rod, *r* is the motor radius, and *L* is the length of the motor center to the initial position of the piston.

**Figure 4:**
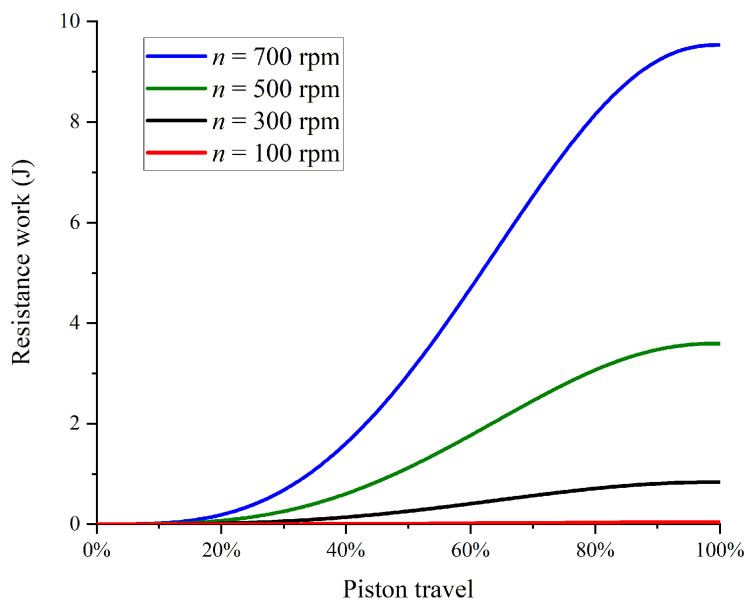
Relation of resistance work and piston travel at different speeds.

As shown in Figure 4, the relation of the resistance work between the porous media and the fluid varies when the piston travels at different speeds, where the compression ratio is 7, the porous media is a copper foam with a specific area of 2980 m^−1^, with a porosity of 0.92, and the fluid is water [28,29].

In the first half of the compression stroke, the porous media is not fully immersed in water and the resistance work is relatively small. With more porous media immersed, the resistance work increases significantly. When the porous media is completely immersed in water, the increase in the resistance work is approximately linear. When the piston reaches 90% of the stroke, the piston gradually approaches the bottom dead center, the compression speed gradually decreases, and the growth rate of the resistance work slows down.

The resistance work can be calculated according to Equations (5) and (9). As shown in Figure 4, when the compression stroke ends and the speed increases from 100 rpm to 700 rpm, the resistance work increases from 0.04 J to 9.54 J; that is, in the compression stroke of the piston, the speed increases by 7 times, while the resistance work increases by 238.5 times. Therefore, increasing the compressor speed significantly increases the flow resistance work between the porous media and the fluid.

**Figure 5:**
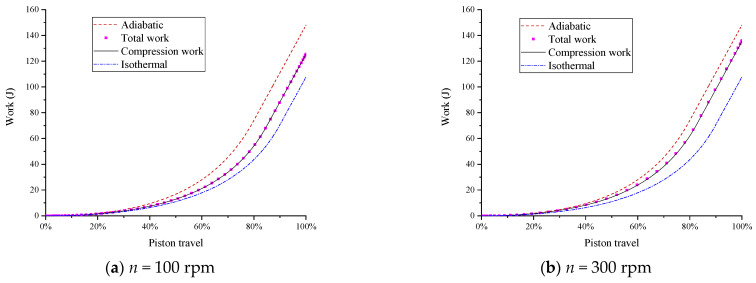

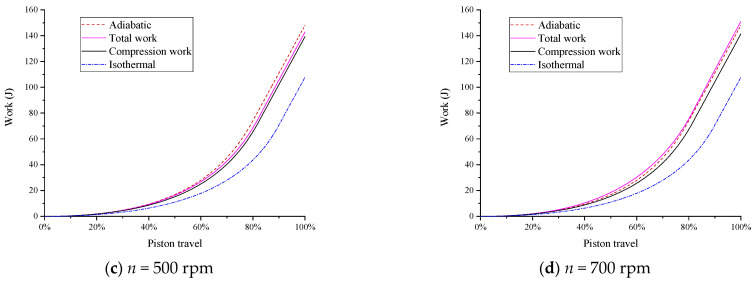
Relation of work and piston travel at different rotational speeds.

Figure 5 shows the variation curves of work with piston strokes at different rotational speeds. It can be seen that the compression work increases as the rotational speed increases. The isothermal and adiabatic compression work are 107.74 J and 148.05 J, respectively, as the compression stroke ends. As shown in Figure 5a, when the rotational speed is 100 rpm, the compression work and total work are 125.48 J and 125.52 J, respectively, at the end of compression stroke. In Figure 5b, when the compression stroke ends at a rotational speed of 300 rpm, the compression work and total work are 135.29 J and 136.13 J, respectively. The compression work and total work increase by 7.82% and 8.45% when the rotational speed increases by 3 times. In Figure 5c, with a rotational speed of 500 rpm, the compression work and total work are 139.34 J and 142.94 J, respectively, at the end of the compression stroke. When the rotational speed increases by 5 times, the compression work and total work increase by 11.05% and 13.88%, while in Figure 5d, when the rotational speed increases to 700 rpm by 7 times, the compression work and total work are 141.56 J and 151.09 J, increases of 12.81% and 20.37%, respectively. For one thing, as the compression speed increases, the time for the piston to complete a compression stroke decreases, thus reducing the heat transfer time between the air and the copper foam, resulting in relatively insufficient air heat transfer and an increase in compression work. For another, the flow resistance increases significantly with the piston speed according to Equation (5). Therefore, the contribution of the flow resistance to the total work also increases with the compression speed. The increase in the total work consists of the increase in the compression work and resistance work, and the resistance work accounts for more as the speed increases.

The resistance also increases as the compression speed increases, which increases the total work as a result. As depicted in Figure 5d, when the rotational speed is up to 700 rpm, the total work (151.09 J) is larger than the adiabatic compression work (148.05 J). That is, the resistance work (9.56 J) is larger than the saved work (6.49 J) due to the heat transfer. The saved work due to the heat transfer is the difference in the compression work between the adiabatic and isothermal piston, as depicted in Equation (15).

(15) $$\Delta W=W_{adi}-W$$

where Δ*W* is the saved work due to the heat transfer, *W_adi_* is the adiabatic compression work, and *W* is the compression work of isothermal piston. The flow resistance between the porous media and the liquid limits the maximum compression speed.

Figure 5 also reflects the relative magnitude of the resistance work to the compression work based on Equations (6) and (9). When the rotational speed is relatively low, such as 100 rpm (Figure 5a) and 300 rpm (Figure 5b), there is little difference between the compression work and the total work, only 0.04 J and 0.84 J, respectively, and the resistance work accounts for 0.034% and 0.62% of the compression work, respectively. That is, the resistance work accounts for a small proportion of the compression work at relatively low rotational speeds. When the rotational speed is relatively high, such as 500 rpm (Figure 5c) and 700 rpm (Figure 5d), the differences between the compression work and the total work is obvious, 3.60 J and 9.54 J, respectively, accounting for 2.58% and 6.74% of the compression work, respectively. That is, the resistance work accounts for a larger proportion of the compression work at relatively high rotational speeds. On the one hand, with the increase in rotational speed, the upper limit of the compression work is under an adiabatic condition. On the other hand, increasing the rotational speed significantly increases the resistance work. Therefore, increasing the rotational speed increases the ratio of resistance work to compression work.

**Figure 6:**
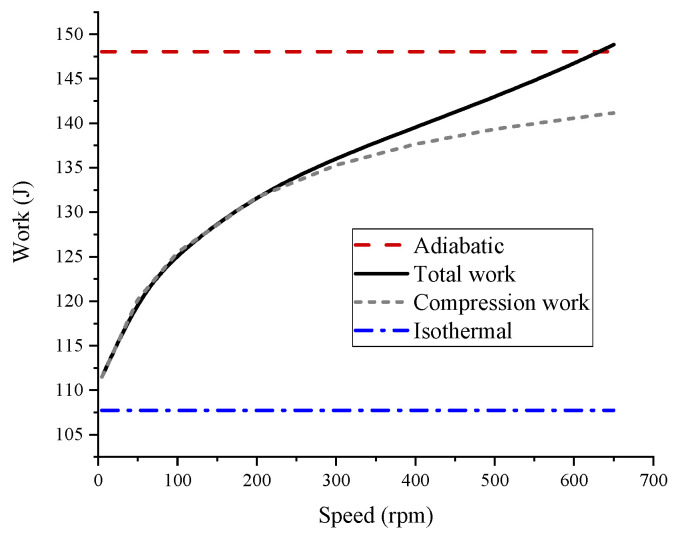
Relation between total work and rotational speed.

The relation between work and rotational speed is shown in Figure 6. According to Equation (10), the compression work and total work increase with the rotational speed nonlinearly. When the rotational speed is below 100 rpm, the increase rates of the compression work and the total work are approximately linear. When the rotational speed exceeds 100 rpm, the increase rates of the compression work and total work decrease gradually. It should be noted that when the rotational speed is below 200 rpm, the increase rates of the compression work and the total work are nearly the same. When the rotational speed is larger than 200 rpm, the increase rate of the total work becomes larger than that of the compression work. It reflects that the influence of resistance becomes more prominent with the increase in rotational speed. When the rotational speed is up to 650 rpm, the total work is equal to that of adiabatic. If the speed continues to increase, the total work will exceed the adiabatic work, and there is no significant energy saving. Therefore, for the isothermal piston, the upper limit of rotational speed is 650 rpm at a compression ratio of 7.

**Figure 7:**
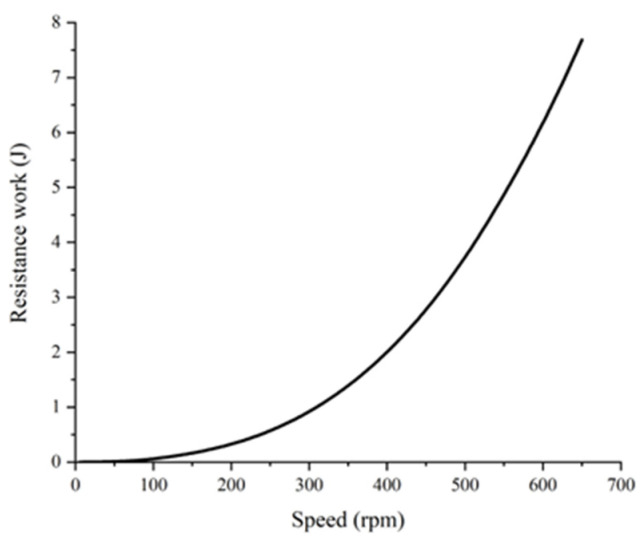
Relation between resistance work and rotational speed.

Figure 7 shows how the resistance work varies with the rotational speed. The increase rate of the resistance work increases with the increase in rotational speed. It can be verified that the proportion of resistance work to total work increases significantly with the increase in rotational speed. When the rotational speed reaches the upper limit of 650 rpm, the resistance work is 7.69 J; that is, the reduced compression work due to heat transfer is 7.69 J.

### 5.2. Energy Conservation Analysis

**Figure 8:**
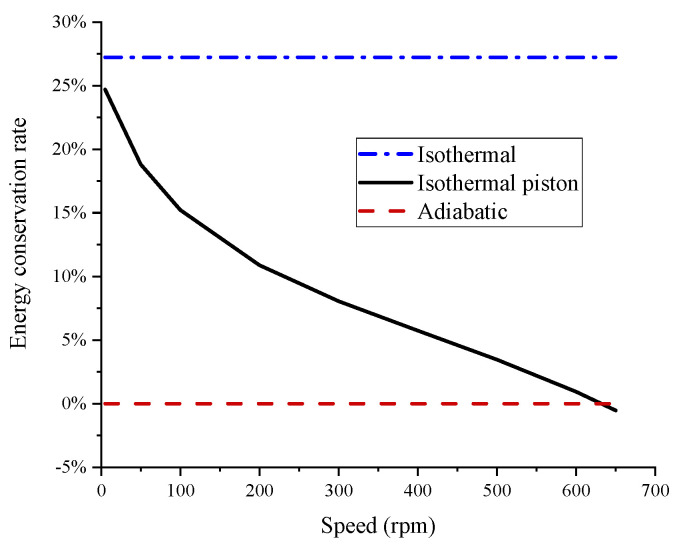
Relation between energy conservation rate and rotational speed.

Considering the flow resistance between the porous media and the liquid, the total work and adiabatic work should be compared to judge the energy saving effect of the isothermal piston. Figure 8 shows the relation between the energy conservation rate of the isothermal piston and rotational speed. Increasing the rotational speed will increase the total work, and then reduce the energy conservation rate of the isothermal piston. According to Equation (11), the maximum energy conservation rate is 27.23%. The energy conservation rate decreases as the rotational speed goes up, and the decrease rate slows down with the rotational speed. When the rotational speed is up to 650 rpm, the energy conservation rate is 0; that, is the total work is equal to the adiabatic work and the compression effect is equivalent to adiabatic compression. The rotational speed should not exceed 650 rpm in order to make the isothermal piston save energy compared with adiabatic compression.

**Figure 9:**
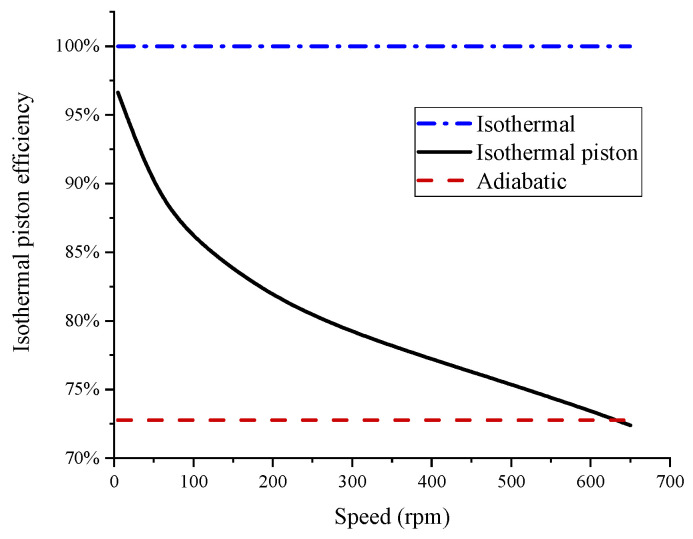
Relation between isothermal piston efficiency and rotational speed.

The relation between the isothermal piston’s efficiency and rotational speed is shown in Figure 9. According to Equation (12), the efficiency of isothermal compression is 100%, while that of adiabatic compression is 72.77%. Increasing the rotational speed lowers the compression efficiency of the isothermal piston. The isothermal piston’s efficiency decreases approximately linearly as the rotational speed is lower than 100 rpm. When the rotational speed is greater than 100 rpm, the decrease rate slows down gradually with the rotational speed. When the rotational speed is larger than 650 rpm, the resistance work exceeds the saved compression work and the compression efficiency is lower than that of adiabatic compression.

It can be concluded from the above analysis that the upper rotational speed of the compressor with the isothermal piston is 650 rpm at a compression ratio of 7, and the lower the rotational speed, the more beneficial to energy saving.

## 6. Conclusions

Up to now, most air compressors operate under adiabatic conditions. Since nearly no heat is transferred to the outside, adiabatic compression requires the most power consumption. Even if the heat can be stored, there are exergy losses due to the poor thermal efficiency. Isothermal compression requires the least energy consumption and the highest energy efficiency because the compression heat is transferred immediately during the compression phase. The isothermal piston is a promising way to lower the compression work, which can realize the near-isothermal compression. The compression heat can be rapidly transferred from the gas to the liquid by maximizing the heat transfer area through a porous media. However, the isothermal piston may bring flow resistance and its effect cannot be neglected at a relatively high speed. In this study, how the rotational speed influences the isothermal piston’s performance was analyzed. The key findings can be summarized as follows:

(1) Increasing the compressor speed significantly increases the resistance work. Within the piston stroke, if the rotational speed is increased by 7 times, the resistance work is increased by about 239 times.
(2) The upper limit of the compression work is the adiabatic work. With the increase in rotational speed, the proportion of resistance work in the total work increases gradually and sharply.
(3) The higher the rotational speed, the greater the increase rate of resistance work.
(4) The total work of the isothermal piston compressor cannot be greater than the adiabatic work. Increasing the rotational speed reduces the energy conservation rate and efficiency.
(5) The maximum rotational speed is 650 rpm at a compression ratio of 7.

## Data Availability

Data is unavailable due to privacy.

## Nomenclature

*C_c_*	Inertia coefficient of porous medium(m^−1^)	Greek symbols	
*C_v_*	Specific heat at constant volume (J∙kg^−1^∙K^−1^)	*ε*	Porosity (%)
*d_p_*	Equivalent spherical particle diameter (m)	ζ	Energy conservation rate
*dp_liq_*	flow pressure of the liquid during the porous media	*η*	Isothermal piston efficiency
*h*	Heat transfer rate [W/(m^2^∙°C)]	κ	Adiabatic exponent
*K*	Permeability of porous medium (m^2^)	*μ*	Dynamic viscosity (Pa∙s)
*L*	Length of motor center to the initial position of the piston (m)	*p*	Density of fluids (kg/m^3^)
*l*	Length of the porous media (m)		
*m*	Mass (kg)	Subscripts and Superscripts	
*p*	Pressure (Pa)	0	Initial condition
*R*	Gas constant (J/kg∙K^−1^)	*adi*	Adiabatic condition
*r*	Motor radius (m)		
*S_v_*	Specific surface area (m^−1^)	*cy*	cylinder
*T*	Temperature (K)	*exp*	Experiment
*u*	Relative velocity between a fluid and a porous medium (m/s)	*iso*	Isothermal condition
*V*	Volume (m^3^)	*liq*	Liquid
*W*	Work (J)	*por*	Porous
*x*	Piston travel (m)		
